# Choosing the Right Tewl Device: A Comparison of Inter‐ and Intra‐Device Reliability of the Vapometer and Dermalab across Measurement Ranges

**DOI:** 10.1111/srt.70358

**Published:** 2026-05-05

**Authors:** Tanja Klotz, Suzanne Edwards, Guy Maddern, Marcus Wagstaff

**Affiliations:** ^1^ Faculty of Health and Medical Sciences Adelaide University Adelaide South Australia Australia; ^2^ Department of Occupational Therapy Royal Adelaide Hospital Adelaide South Australia Australia; ^3^ School of Public Health Adelaide University Adelaide South Australia Australia; ^4^ Basil Hetzel Institute for Translational Health Research Queen Elizabeth Hospital Woodville South Australia Australia; ^5^ Department of Plastic and Reconstructive Surgery Royal Adelaide Hospital Adelaide South Australia Australia; ^6^ Adelaide Medical School Adelaide University Adelaide South Australia Australia

**Keywords:** evaporimeter, insensible water loss, skin, scar, transepidermal water loss

## Abstract

**Background:**

Measurement of transepidermal water loss (TEWL) is widely used to evaluate skin barrier function.

**Materials and Methods:**

This study compared two devices—the DermaLab (DL) TEWL probe and the VapoMeter (VM) — from data collected across three studies to measure TEWL in scarred and normal skin. Each site was measured three times per device, alternating devices to allow vapor clearance. Inter‐device reliability was assessed from 1,617 paired locations, each averaged from the three readings. Intra‐observer reliability was able to be determined from a dataset of 1,628 DL and 1,635 VM measurements.

**Results:**

Data were stratified into low, medium, and high TEWL ranges based on measurement distribution and observations. Overall inter‐device reliability was ‘good’ but varied by range: ‘poor’ in low, ‘excellent’ in medium, and ‘moderate’ in high TEWL ranges. In the medium range, the VM reported nearly twice the TEWL values compared to the DL, while in the high range, it read approximately 34% higher.

**Conclusions:**

Both devices showed excellent intra‐observer reliability overall. However, in the low TEWL range, the DL exhibited ‘moderate’ reliability, whereas the VM showed slightly better, ‘good’ reliability. Differences in chamber architecture—semiopen for the DermaLab and closed for the VM—appear to drive the observed variability in measurement reliability across TEWL ranges. Analysis of this large dataset indicates that the VM likely approaches saturation at the categorized ‘high’ TEWL values, constraining its accuracy, whereas the DermaLab exhibits reduced measurement stability at low TEWL levels, limiting its applicability under those conditions. Targeted methodological work is needed to refine DL performance in the lowTEWL regime and to more precisely define the upper operational limit of the VM.

## 1 Introduction

The outermost layer of the epidermis, the stratum corneum, plays a critical role in maintaining the skin's barrier function. It is composed of dead, flattened, keratin‐rich corneocytes, which are embedded in a complex mixture of intercellular lipids, including ceramides, free fatty acids, cholesterol, and cholesterol sulfate [1, 2]. The measurement of transepidermal water loss (TEWL) serves as an indicator of the integrity and effectiveness of this barrier [1].

Water primarily diffuses through the intercellular spaces of the stratum corneum, making TEWL a useful parameter for assessing skin barrier function [3]. In research settings, TEWL measurements are employed to evaluate the effects of various products (e.g., chemicals [4], irritants [5], creams [6]) and conditions (e.g., endogenous [7] or environmental factors [8]) on the skin. Evaporimeter devices are widely used to quantitatively measure the rate of TEWL in both human and animal studies.

However, TEWL measurement is known to exhibit high variability. Consequently, standardized methodologies have been recommended by multiple guidelines to ensure consistency and reliability of results [9, 10, 11]. In addition to intra‐experimental variability, inter‐device differences also pose challenges. A systematic review of the measurement properties of TEWL devices found frequent correlations between instruments, but highlighted significant limitations in study design and statistical methodology across the literature [12].

Evaporimeter devices are available in closed, open, and semi‐open chamber designs. These instruments typically consist of a chamber containing a series of humidity sensors positioned a few millimeters above the skin during use. The DL TEWL probe consists of two sets of temperature and humidity sensors mounted at different heights above the skin surface [13]. The rate of TEWL is based on Nilsson's Vapor Pressure Gradient method and Fick's Law of Diffusion measured by changes in water vapor density between the sensors and is expressed in grams of water per square meter per hour (g/m^2^/h) [13]. The VM also contains sensors for temperature and relative humidity. The TEWL is calculated from the rate of linear increase in relative humidity within the chamber after placing it on the skin and us is also expressed in g/m^2^/h units [14].

Each device type has its own advantages and limitations. The VM (Delphin Technologies, Kuopio, Finland) is a closed‐chamber device that, due to its design, can eliminate external air currents and theoretically reduce measurement error. However, the accumulation of humidity within the chamber may affect the rate of water evaporation from the skin. As humidity builds up, the device is unsuitable for continuous TEWL measurements, which may be necessary in certain research protocols. Reported measurement error for the VM across 11 studies ranged from 4.7% to 76.9% [12]. In comparison, the DL (Cortex Technologies, Hadsund, Denmark), a semi‐open chamber design, incorporates a mesh layer that protects against ambient air flow while preventing humidity buildup. The reported measurement error for the DL ranged from 0% to 20.75% across six studies [12]. In a study by Anthonissen et al. [15] the DL measuring the TEWL of scars and normal skin was reported to have intraclass correlation coefficients (ICC) for intra‐observer reliability of 0.86–0.88 [15]. This study also calculated measurement error with the standard error of the mean (SEM), resulting in SEM values of 1.24–1.74 [15].

Two studies have directly compared the VM and DL devices. In the first, Cohen et al. [16] evaluated measurements obtained from a test substrate positioned at 0°, 45°, and 90° angles [16]. The results showed that, for both devices, the measured rate of water loss was significantly lower at 45° and 90°, indicating that the angle of device placement affects TEWL readings. This finding challenges the manufacturers claim that closed‐chamber devices yield measurements “unaffected by the instrument's orientation” [17].

In the second study, Fluhr et al. [18] compared multiple TEWL measurement devices using in vivo human and mouse models. The VM and DL demonstrated strong correlation, with Pearson correlation coefficients of 0.9716 in mice (*n* = 68) and 0.9050 in humans (*n* = 22). While the mouse model included a robust number of paired measurements, the sample size for the human model was relatively limited (*n* = 22) [18].

At our center, it was observed that the DL and VM devices often produced differing TEWL readings. Notably, the DL appeared to yield more variable measurements at low TEWL levels. In the medium range, the VM frequently produced values nearly twice as high as those recorded by the DL. However, at high TEWL levels, the two devices tended to yield convergent readings, suggesting that the VM may be approaching saturation in this range, potentially due to its closed‐chamber design.

The primary aim of this study is to compare the VM and DL using a large dataset by evaluating reliability through ICC and measurement error (or agreement) via limits of agreement (LoA), and coefficient of variation (CV)—as recommended by Mokkink et al. [19]. These statistical approaches are specifically suited for continuous data and align with the study design [19].

Additionally, leveraging the extensive paired dataset from the VM and DL, a secondary aim is to assess how well the devices correlate across low, medium, and high TEWL ranges. This will help determine whether the observed variations in measurement are consistent and detectable. The findings aim to inform clinicians and researchers in selecting the most appropriate device based on their specific measurement needs.

## 2 Materials and Methods

### 2.1 Design

This comparative study utilized convenience sampling, drawing participants from three separate research protocols. In each study, the same technique of parallel in vivo measurements of human skin TEWL were performed using both a single VM device and DL device.

The devices were alternated in rapid succession at the same anatomical site, thereby minimizing environmental variation. However, each device was adequately ventilated before re‐measuring. For each site, three TEWL measurements were taken using each device, and the mean value was used for data analysis. The process of measuring a single test site for all studies was as follows:

DL1→VM1→DL2→VM2→DL3→VM3
DermaLab TEWL Result = (DL1 + DL2 + DL3) / 3
VapoMeter TEWL Result = (VM1 + VM2 + VM3) / 3

Therefore, the paired DL and VM measurements were obtained simultaneously at the same anatomical site, ensuring direct comparison.

In Study 1, all measurements were taken by the primary author. In Studies 2 and 3, approximately 95% of the measurements were taken by the primary author, with the remaining measurements conducted by two co‐authors. This ensured consistency of measurement technique.

#### 2.1.1 Study Cohorts

- Study 1 evaluated scar outcomes following CO_2_ ablative laser treatment in burn patients. TEWL measurements were obtained from pre‐lasered and post‐lasered scars, as well as from non‐lasered control scar sites. Thus, all measurements in this study were performed on scarred skin. This study resulted in 195 areas that were measured by both devices, comprising 12% of the data for this study. Environmental conditions were recorded at each data collection event. Data was collected from June 2020‐March 2023, however, due to study commencement delays the utilization in the early stages was minimal. Much of the data was collected in the late stage of the study.
- Study 2 assessed TEWL in active burn scars and anatomically matched contralateral sites of clinically normal skin. This study resulted in 162 areas that were measured by both devices, comprising 10% of the data for this study. Environmental conditions were recorded at each data collection event. Data was collected from March 2022‐April 2024.
- Study 3 involved tape‐stripping nine designated areas on the volar forearms of healthy volunteers, with one additional unstripped control site. Tape stripping involved 15 sequential strips of D ‐Squame (CuDerm Corp., Dallas, TX, U.S.A.) 22 mm discs with an application pressure of 2N for two seconds. Eight of the tape‐stripped areas received topical product applications. TEWL was measured at baseline and then hourly for four hours following product application. For this study, environmental measurements were recorded only at baseline time points, that is when the first TEWL measurements were taken. This was due to measurements occurring in the same closed, air‐conditioned room providing consistent conditions throughout. For more details see Klotz et al. [6]. This study resulted in 1260 areas that were measured by both devices, comprising 78% of the data for this study. Data was collected from July 2023 to July 2024.

#### 2.1.2 Ethics and Governance

All three studies received ethics and governance approval through the expedited review process of the *Central Adelaide Local Health Network (CALHN) Human Research Ethics Committee (HREC)* and CALHN Research Services. Each study was approved in accordance with the National Health and Medical Research Council (NHMRC) National Statement on Ethical Conduct in Human Research (2007), incorporating all updates. The ethics approval reference numbers were:

- HREC/18/CALHN/361 (18 February 2021)—CO2 ablative laser outcomes study
- HREC/21/CALHN/15640 (24 November 2021)—Scar measurement study
- HREC/21/CALHN/16923(25 January 2023)—Tape‐stripping effect of moisturizers study

#### 2.1.3 Participants

A total of 99 participants (49M:50F, aged 18–84 years) were enrolled across the three studies after providing written informed consent. This yielded 1617 paired TEWL measurement sites for inter‐device reliability, distributed as follows:

- 276 from burn scars (Study 1: *n* = 195 Study 2: *n* = 81)
- 231 from normal skin (Study 2: *n* = 81, Study 3: *n* = 150)
- 1,110 from tape‐stripped sites (Study 3: *n* = 1110)

#### 2.1.4 Measurement Protocol

Each site was measured using both the VM and the DL. All measurements were conducted by the same investigator at each individual site to ensure consistency. A single investigator performed the majority of measurements across all three studies.

**FIGURE 1:**
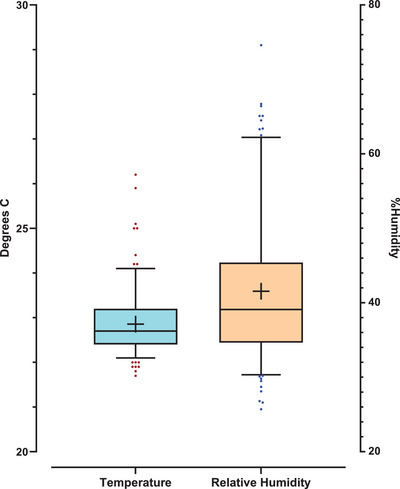
Temperature (left, light blue) and relative humidity (right, orange) conditions of 193 occurrences of TEWL measurement. Mean temp 22.9°Celsius, standard deviation 0.68. Mean relative humidity 41.5%, standard deviation 9.46%. The box indicates the interquartile range, the median is the line inside the box, the mean is indicated with a ‘+’ symbol and the edges of the whiskers extend from 5%–95% spread of data.

All measurements were conducted in the same temperature‐controlled room in the Burns Unit at Royal Adelaide Hospital, South Australia, to minimize environmental variability. Environmental temperature and relative humidity were recorded at the time of each measurement using the DL TEWL probe. A total of 193 environmental readings were obtained across all studies. In the tape‐stripping study, readings were only collected at baseline time points. Temperature and humidity was generally well controlled with a mean of 22.9°C (*SD* = 0.68°C) and humidity mean of 41.5% (*SD* = 9.46%) as shown in Figure 1.

Participants with burn scars were instructed to remove pressure garments and complete any other required assessments before TEWL measurement were taken. For study 3, participants completed background data collection, the consent form, and the marking of test sites prior to commencing TEWL measurements. This allowed time for acclimatization to room temperature before device application.

Measurement sites were delineated on the skin using pen to define an area of approximately 2 cm × 2 cm, ensuring accurate and consistent repositioning of both devices. The TEWL measurement process took approximately 3 min per site, with devices alternated to allow each chamber to clear and re‐equilibrate with ambient conditions between readings. The order in which device was first was not consistent nor was it formally randomized, however, for the majority of measurements the DL was first then the VM.

The DL device was configured with a time target of 30 s and a standard deviation (SD) target of 0.2. If the SD target was not reached within 30 s, the measurement defaulted to the 30‐s time limit. The standard adapter was used with the VM for all measurements.

All results were recorded manually on structured data collection sheets and later transferred to a digital spreadsheet for analysis.

#### 2.1.5 Recruitment and Measurement Scheduling

Participants in the laser outcomes study were scheduled according to the predefined treatment timeline. Those in the scar activity study were recruited during routine outpatient follow‐up appointments at the burns unit. For both studies, participants were individuals with a history of burn injury. In the laser study, only scarred sites were assessed, whereas in the scar activity study, both scarred and site‐matched normal skin were evaluated.

Volunteers for the moisturizer evaluation study were healthy individuals without burn injuries or skin conditions. Each participant had nine sites tape‐stripped, with moisturizers applied to eight of them, and one non‐tape‐stripped control site. TEWL was measured hourly over four hours following baseline measurements.

### 2.2 Devices

The DL Combo (Cortex Technologies, Hadsund, Denmark) comprises a base unit, a wirelessly connected laptop running DL SkinLab software (version 2.1.1.5), and supports multiple interchangeable probes. For this study, the DL TEWL probe, a semi‐open chamber with a diameter of 1.1 cm, was utilized. This probe measures the water evaporation gradient from the skin surface using two pairs of sensors. To maintain infection control, disposable plastic probe protectors were replaced between participants.

Measurements were recorded in units of g/m^2^/h. The system allows for two measurement settings: a time‐based target (ranging from 1 to 1200 s), or a SD target (range 0.10–10.0), where measurement ends once the TEWL reading stabilizes. The default SD target is 0.2; however, when this target is not reached—particularly at low TEWL levels—automatic stopping does not occur. To address this, a time target of 30 s was also implemented to ensure measurements concluded even when the SD criterion was unmet. Re‐measurement was performed after an approximately one‐minute interval, with careful monitoring of the initial TEWL value. If an elevated starting value was observed, the measurement was cancelled and additional ventilation time was allowed to ensure a fully cleared chamber prior to repeat measurement. TEWL was also assessed using the VM SWL5124 (Delfin Technologies Ltd., Kuopio, Finland), a closed‐chamber, handheld, battery‐operated device. All measurements employed the standard adapter, which has a 1 cm diameter chamber opening containing internal humidity sensors. During measurement, the device monitors the increase in relative humidity inside the chamber and automatically calculates the evaporation rate (g/m^2^/h) [17]. The measurement range is reported as 3–200 g/m^2^/h, with measurement durations between 5–16 s using the standard adapter [17]. Unlike the DL, the VM does not support configurable time or SD targets. Although the device can display environmental and relative humidity, these parameters were more conveniently monitored via the DL laptop interface. The VM indicates when the chamber has completed ventilation and is ready for re‐measurement, allowing efficient alternation with the DL.

Both devices were acquired just prior to the research studies being undertaken and had not been utilized in other studies. They were therefore considered to be adequately factory calibrated at the time of data collection. The VM has a twoyear factory calibration guarantee and was used predominantly within this period (2022–2024). Recommended calibration intervals for the DL probe are variable and depend on usage and environmental conditions. Both devices were stored in clean, dry conditions, handled carefully, and subjected to routine functional checks; under these circumstances, they were considered to be performing consistently and providing stable measurements.

### 2.3 Statistical Analysis

All data analyses were performed using Stata Statistical Software: Release 15.1 College Station, TX: StataCorp LP and IBM SPSS Statistics for Windows Software (IBM Corp, 2023, Version 29.0.1.0 Armonk, NY, USA). Calculation of the standard error of measurement was calculated in excel using the formula: SEM = SD*(SQRT(1‐ICC)), where SD was the calculated SD using SPSS and ICC values as calculated by the statistician using Stata.

Bland–Altman plots were generated in Excel. The LoA were calculated as the mean difference ± 1.96× the SD.

#### 2.3.1 Inter‐Device and Intra‐Observer Reliability

ICC's Were Performed Using a Two‐way Mixed‐effects With Consistency of Agreement ICC(3, 1) Method

#### 2.3.2 ICC Interpretation

ICC values were interpreted according to the criteria described by Koo and Li [20], as follows:

Poor: 0‐< 0.5
Moderate:0.5‐< 0.75
Good: 0.75‐< 0.9
Excellent:≥0.9

#### 2.3.3 Sensitivity Analysis

Statistics were recalculated using cutoff values one unit higher and one unit lower than those originally defined by the DL for the low, medium, and high TEWL ranges (see Appendix 1, Tables A1 and A2).

## 3 Results

### 3.1 Inter‐Device Reliability

**FIGURE 2:**
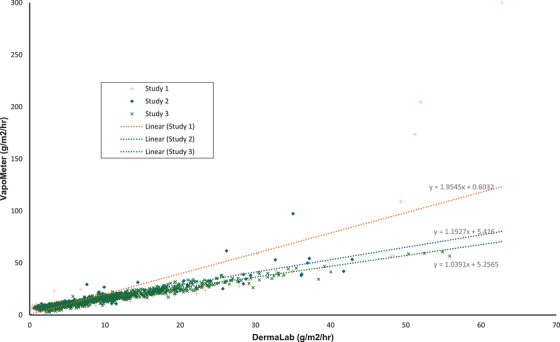
Scatterplot of all raw values from the three studies as measured by the DermaLab and VapoMeter at the same location, *n*=1617. Each measurement is the averaged value of three measurements at the same location by each device. Linear trendlines of each study's data are shown with dotted lines.

A scatterplot of all 1,617 paired data points from the three different studies —where both the DL and VM were used at the same skin site—demonstrates that the TEWL measurements obtained from the two devices positively correlate and appear to have a linear relationship (Figure 2). Four of the apparent outliers at high TEWL originated from Study 1 (orange ‘+’ symbol), skewing the linear relationship upwards.

**FIGURE 3:**
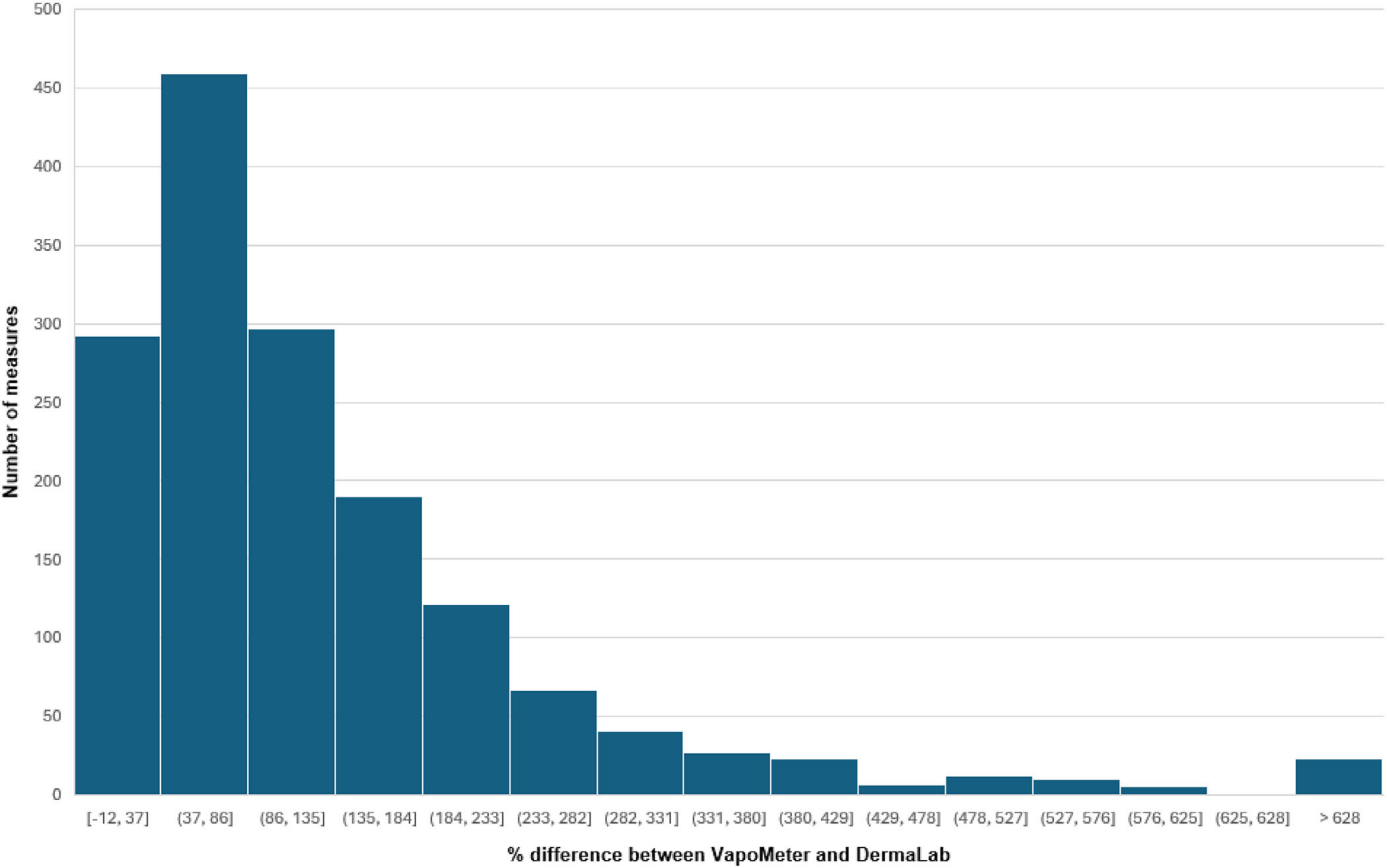
Histogram of percentage difference between measures taken by the VapoMeter and DermaLab at the same location *n*=1612, five outliers have been removed. Most of the measures (*n*=459, 28% of the data) with the VapoMeter were between 37%–86% greater than the DermaLab measurements. Percentage difference = (VapoMeter value—DermaLab Value / DermaLab Value)x100.

**FIGURE 4:**
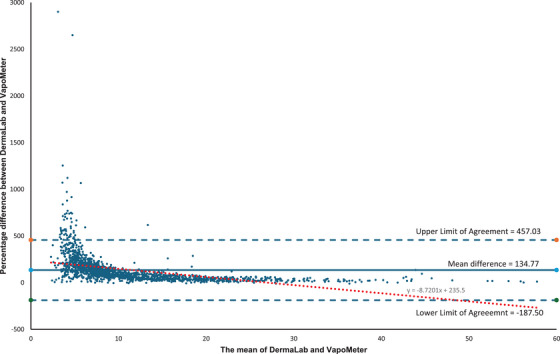
Bland‐Altman plot of the paired data when measurements were taken at the same site by the DermaLab and VapoMeter. The percentage difference between the DermaLab and VapoMeter is graphed against the mean of the DermaLab and VapoMeter readings. Percentage difference = (VapoMeter value—DermaLab Value / DermaLab Value) x100.

The percentage differences between the VM and DL measurements—excluding five of the outlier data points (*n* = 1,612)—shows that the VM reported TEWL values 37%–86% higher than the DL in 28% of cases (*n* = 459) (Figure 3). A Bland–Altman plot of the mean percentage difference of all the data shows the difference between the devices is typically substantially higher at 135% (*SD* = 164%, media*n* = 92%, LoA: −187.50 to 457.03) and that most data points fall within the LoA, suggesting reasonable concordance between the two devices (Figure 4). Intraclass correlation coefficient (ICC) analysis indicated ‘good’ reliability across the full dataset (ICC = 0.8383; 95% CI: 0.8216–0.8535) (Table 1).

**TABLE 1:** Intraclass correlation coefficients (ICC) and 95% confidence intervals (CI) for inter‐device reliability where measurements were taken with DermaLab and the VapoMeter. Statistics are presented for all data and the three ranges of low, medium and high TEWL. The mean percentage difference, standard deviation (SD), coefficient of variation (CV) and Standard Error of Measurement (SEM) are calculated using the percentage difference between the two devices.

Sample	Mean % difference^a^	n	SD	CV	ICC (95% CI)	Interpretation	SEM
All data	134.77%	1617	162.76%	120.77%	0.8383^*^ (0.8216, 0.8535)	Good reliability	65.45%
Low range^b^	325.57%	339	261.67%	80.37%	0.3156^*^ (0.1527,0.4472)	Poor reliability	216.48%
Medium range^c^	97.78%	1006	53.04%	54.24%	0.9088^*^ (0.8968, 0.9194)	Excellent reliability	16.02%
High range^d^	33.74%	272	35.59%	105.48%	0.6143^*^ (0.5103, 0.6962)	Moderate reliability	22.10%

^a^ *Percentage difference = (VapoMeter value—DermaLab Value / DermaLab Value) x100.

^b^ Low range: DermaLab 0–3; VapoMeter 0–8 (approximately).

^c^ Mid range: DermaLab > 3 – 15; VapoMeter > 8–20 (approximately).

^d^ High range: DermaLab > 15; VapoMeter > 20 (approximately).

^*^ *p*<0.001.

**FIGURE 5:**
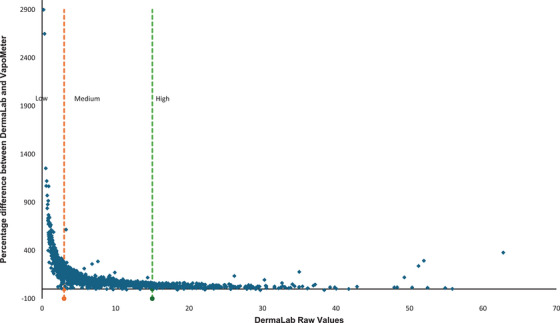
Graph used to determine arbitrary low, medium and high TEWL ranges for paired data where VapoMeter and DermaLab measured the same location. Percentage difference = (VapoMeter value—DermaLab Value / DermaLab Value)x100.

Figure 5 presents a plot of DL values against the percentage difference between the two devices, enabling the delineation of arbitrary low, medium, and high TEWL ranges. Plotting the percentage difference against VM values yielded a similar distribution. The greatest variability was observed in the low range, defined as < 3 g/m^2^/hr when measured by the DL. The medium range, defined as 3–15 g/m^2^/hr, showed a tapering of variability, while the high range, > 15 g/m^2^/hr, demonstrates more consistent agreement.

Extrapolation of these arbitrary cut‐off values to the paired dataset therefore yields the following range definitions:

- Low range: DermaLab 0‐< 3 g/m^2^/hr, VapoMeter 0–8 g/m^2^/hr, *n* = 339
- Medium range: DermaLab ≥3–15 g/m^2^/hr, VapoMeter > 8–20 g/m^2^/hr, *n* = 1006
- High range: DermaLab > 15 g/m^2^/hr, VapoMeter > 20 g/m^2^/hr, *n* = 272

#### 3.1.1 Low Range Measurements

**FIGURE 6:**
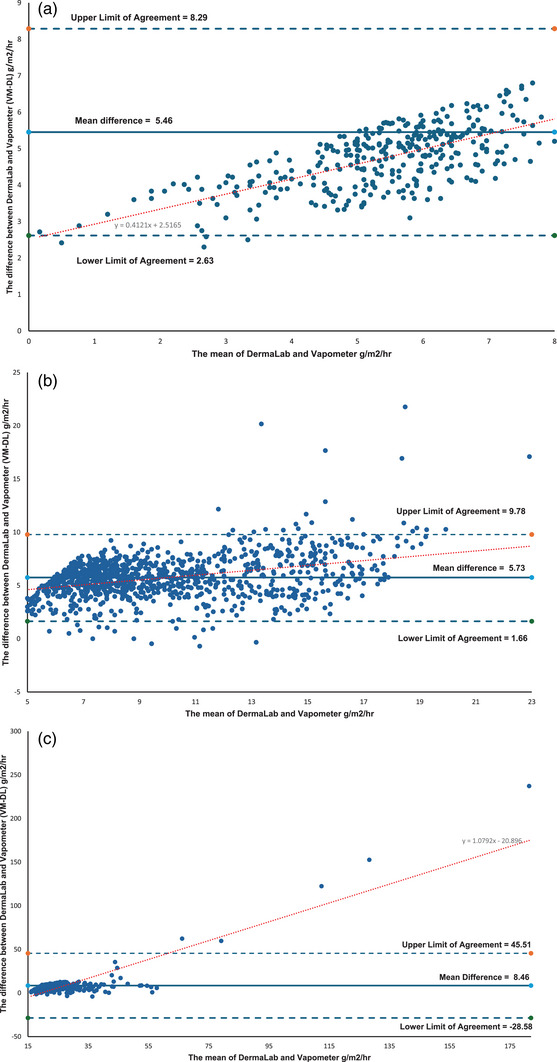
(a) Bland‐Altman Plot of paired DermaLab and VapoMeter low range data which equates to DermaLab measuring 0–3 g/m2/hr and VapoMeter measuring 0–8 g/m2/hr (*n*=339). At lower TEWL measurements the difference between the VapoMeter and DermaLab trends toward the lower Limit of Agreement. Proportional bias is indicated by the linear regression line. (b) Bland‐Altman Plot of paired DermaLab and VapoMeter Medium range data which equates to DermaLab measuring > 3–15 g/m2/hr and VapoMeter measuring > 8–20 g/m2/hr (*n*=1006). At the lower half of TEWL measurements in this range the difference between the VapoMeter and DermaLab is more clustered around the mean and the difference between the devices becomes more variable in the upper half of this data range. Proportional bias is indicated by the linear regression line. (c) Bland‐Altman Plot of paired DermaLab and VapoMeter High range data which equates to DermaLab measuring > 15 g/m2/hr and VapoMeter measuring > 20 g/m2/hr (*n*=272). At very high TEWL there are five outliers where the VapoMeter has measured much greater TEWL than the DermaLab. Proportional bias is indicated by the linear regression line.

Statistical details for the paired data—where measurements from the same test site were obtained using both the VM and DL—are presented in Table 2. For the low‐range subset of paired data (*n* = 339), the Bland–Altman plot (Figure 6a) shows there is a consistent positive bias, indicating that the DL consistently produces TEWL readings above that of the VM. A mean difference of 5.46 g/m^2^/hr between the VM and DL, with LoA ranging from 2.63 to 8.29 g/m^2^/hr containing most of the data points (Figure 6a). There is a proportional bias for the low range data indicating that as the mean TEWL values increase so does the difference between the DL and VM, as shown by the linear regression line in Figure 6a. Examination of the DL values in the low range showed high variability (CV 31.7%) compared to moderate variability demonstrated by the VM measures (CV 19.5%) (Table 2).

**TABLE 2:** Statistical properties of the paired data used in the inter‐device analysis. This includes all data, low, medium and high range categories as previously defined for each device.

Device—range	n	Mean (g/m2/hr)	Standard error of the mean (g/m2/hr)	Standard Deviation (g/m2/hr)	Coefficient of Variation
DermaLab—All data	1617	8.678	0.198	7.982	92.0%
DermaLab—Low	339	2.040	0.035	0.647	31.7%
DermaLab—Medium	1006	7.047	0.101	3.188	45.2%
DermaLab—High	272	22.986	0.511	8.438	36.7%
Vapometer—All data	1617	14.813	0.321	12.910	87.2%
Vapometer—Low	339	7.500	0.080	1.464	19.5%
VapoMeter—Medium	1006	12.772	0.124	3.939	30.8%
VapoMeter—High	272	31.478	1.451	23.927	76.0%

The percentage difference in this low range is highly variable. The mean percentage difference between the VM and DL was 326% (Table 1), with a CV of 81% (Table 1). This high variability corresponds to ‘poor’ reliability, as reflected in an ICC of 0.3156 (95% CI: 0.1527–0.4472) (Table 1)

#### 3.1.2 Medium Range Measurements

In the medium‐range subset of paired data (*n* = 1006), the Bland–Altman plot (Figure 6b) shows that again there is a positive and proportional bias and a mean difference of 5.73 g/m^2^/hr between the VM and DL—similar to that observed in the low range—but with slightly wider (LoA: 1.66 to 9.78 g/m^2^/hr). Within the lower half of this TEWL range, the differences between devices are tightly clustered around the mean. In contrast, variability increases in the upper half of this range. DL values and VM values showed high variability in this range with CV values of 45% and 31% respectively.

Despite this, percentage differences within the medium range remain relatively consistent (Figure 5), with a mean difference of 98% (Table 1) and a lower CV of 54%. This suggests that, on average, VM readings were approximately double those of the DL. Reliability for this range was classified as ‘excellent,’ with an ICC of 0.9088 (95% CI: 0.8968–0.9194) (Table 1).

#### 3.1.3 High Range Measurements

For the high‐range TEWL measurements (*n* = 272), the Bland–Altman plot (Figure 6c) displays a positive and proportional bias and a mean difference of 8.46 g/m^2^/hr, with substantially wider (LoA: –−28.58 to 45.51 g/m^2^/hr). On average, the VM recorded TEWL values 33.74% higher than the DL in this range (Table 1). Variability in the data is observed to lower with the DL (*CV* = 36.7%) compared to the VM (*CV* = 76%). At TEWL values above 50–60 g/m^2^/hr (as measured by either device), the data included apparent outliers, where the VM reported values 50%–250% higher than those from the DL (Figure 6c).

Due to the presence of these outliers and broader variability, reliability in the high range was classified as ‘moderate,’ with an ICC of 0.6143 (95% CI: 0.5103–0.6962) (Table 1). The CV for the percentage difference in this range was 105%, indicating substantial dispersion.

#### 3.1.4 Sensitivity Analysis

Statistics were recalculated using cutoff values one unit higher and one unit lower than those originally defined by the DL for the low, medium, and high TEWL ranges (see Appendix 1, Tables A1 and A2). Adjusting the cutoffs by one unit had minimal effect on the medium and highrange samples. In contrast, shifting the lowrange boundary produced a marked change in reliability: lowering the cutoff by one unit reduced the ICC from 0.3156 (*p* < 0.001; 95% CI: 0.1527–0.4472) to 0.1950 (*p* = 0.098; 95% CI: −0.1188–0.4207), whereas increasing the cutoff by one unit improved reliability to a moderate level (ICC  =  0.5367; *p* < 0.001; 95% CI: 0.4503–0.6095).

### 3.2 Intra‐Observer Reliability

In each study from which data were obtained, the same observer conducted three repeated measurements at each test site using the same device. Measurements were typically alternated between the VM and the DL to allow for water vapor clearance from the test chamber. This protocol enabled calculation of intra‐observer reliability for both devices—for all data and across the pre‐defined low, medium, and high TEWL ranges.

The statistical properties of the datasets used to assess intra‐observer reliability are presented in Table 3. In some instances, only one of the two devices was available, resulting in more data points than in the paired dataset and unequal sample sizes for each device.

**TABLE 3:** Statistical details of data used for intra‐observer analysis. Three measures were taken with each device. The average of the three measures at each site were utilised to categorise in the low, medium and high range as previously defined and to then calculate the statistics below.

Device—range	n	Mean (g/m2/hr)	SEM (g/m2/hr)	Standard Deviation (g/m2/hr)	Coefficient of Variation
DermaLab—All data	1628	8.676	0.6672	8.005	92.3%
DermaLab—Low	343	2.044	0.3958	0.646	31.6%
DermaLab—Medium	1012	7.038	0.5347	3.190	45.3%
DermaLab—High	273	23.078	1.1581	8.446	36.6%
Vapometer—All data	1635	14.762	1.8806	12.916	87.5%
Vapometer—Low	280	6.588	0.4970	1.236	18.8%
VapoMeter—Medium	1047	12.246	0.5898	3.242	26.5%
VapoMeter—High	308	30.746	4.1592	22.623	73.6%

As shown in Table 4, the DL demonstrated ‘excellent’ intra‐observer reliability for all data combined, medium range, and high range TEWL. But was only ‘moderate’ reliability (ICC: 0.6247, 95% CI: 0.5503–0.6886) in the low range. The VM also exhibited ‘excellent’ intra‐observer reliability for all the data, medium and high range, and outperformed the DL in the low TEWL range, where it demonstrated ‘good’ reliability (ICC: 0.8383, 95% CI: 0.8024–0.8685).

**TABLE 4:** Intraclass correlation coefficients (ICC) and 95% confidence intervals (CI) for DermaLab and VapoMeter when measuring each site three times. The values for all the data, and the three ranges of low, medium and high TEWL are shown with the interpretation of the intra‐observer reliability.

Device—range	n	ICC (95% CI)	Interpretation	Significance
DermaLab All data	1628	0.9932 (0.9926, 0.9937)	Excellent	< 0.001
DermaLab Low range (0–3)	343	0.6247 (0.5503, 0.6886)	Moderate	< 0.001
DermaLab Medium range (>=3 – 15)	1012	0.9719 (0.9688, 0.9748)	Excellent	< 0.001
DermaLab High range (> 15)	273	0.9812 (0.9770, 0.9848)	Excellent	< 0.001
Vapometer All data	1635	0.9788 (0.9770, 0.9806)	Excellent	< 0.001
Vapometer Low range (0–8)	280	0.8383 (0.8024, 0.8685)	Good	< 0.001
Vapometer Medium range (>=8 – 20)	1047	0.9669 (0.9632, 0.9702)	Excellent	< 0.001
Vapometer High range (> 20)	308	0.9662 (0.9591, 0.9723)	Excellent	< 0.001

## 4 Discussion

This study presents the largest dataset to date comparing paired measurements from the DL TEWL probe and the VM, allowing not only correlation analysis but also a detailed examination of device performance across this study‐defined ranges of low, medium, and high TEWL. By drawing on data from three separate studies, we were able to conduct a comprehensive evaluation of both intra‐observer and inter‐device reliability. All three studies demonstrated a similar linear trend in the distribution of TEWL values. However, the outliers at high TEWL levels occurred predominantly in Study 1 (CO_2_ laser study), in which only active scars were measured. While these outliers may suggest a more polynomial than linear relationship at the upper end of the scale, the number of data points in this range is insufficient to confidently characterize the behavior of the devices at very high TEWL values.

Both devices demonstrated *good to excellent* intra‐observer reliability. However, inter‐device comparison revealed a consistent positive and proportional bias, with the VM yielding higher TEWL values than the DL. The percentage difference decreased as TEWL increased, indicating convergence of measurements at higher TEWL values prompting analysis by stratified TEWL ranges (low, medium, high).

Device selection should be guided by the expected TEWL range. At low TEWL values, the DL exhibited greater variability—likely due to difficulties meeting the default 0.2 SD threshold within the set 30‐s acquisition window. In this context, the VM may offer more accurate readings. Conversely, at higher TEWL levels, the VM showed a tendency to plateau and produce greater variability, suggesting possible saturation of its closed chamber. In contrast, the DL's semi‐open chamber design appeared better suited for accurately capturing high TEWL values.

This pattern of higher variability in the lowTEWL range was also evident in the sensitivity analysis. Shifting the cutoff between the low and medium TEWL categories by ± 1 unit (based on DL measurements) altered the results for the lowTEWL group, whereas the medium and highTEWL groups were minimally affected. This further supports that variability at the lower end of the TEWL spectrum is more pronounced and more sensitive to classification thresholds. The wide LOA observed in the high TEWL range are partly attributable to five outlier measurements from the VM, which recorded unexpectedly high values where the DL measured greater than approximately 35 g/m^2^/h. Due to the limited number of data points in this range, it remains unclear whether these outliers reflect true physiological readings or measurement artefacts.

Comparisons with previous studies support the validity of our findings. For example, Anthonissen et al. [15]. reported intra‐observer ICCs of 0.78–0.88 for the DL, which aligns with the ICCs observed in our study, though their reported confidence intervals were notably wider. Their study also reported standard error of measurement (SEM) values of 1.24–1.74, while our study achieved lower SEMs (0.40–1.16), likely due to a larger sample size and adherence to the recommended protocol of three repeated measurements per site [9].

Another methodological difference worth noting is the measurement duration: Anthonissen et al. [15] study used a 60‐s acquisition window compared to 30 s in this analysis. While both studies employed the same SD threshold (0.2), using a longer duration may artificially elevate low‐range TEWL values, as TEWL typically increases over time. Previous work suggests TEWL stabilization occurs within 30–45 s, supporting the shorter measurement window used here [10].

Fell et al. [21]. found a linear relationship between the DL and the open‐chamber Tewameter. Similarly, our data suggest a likely linear relationship between the DL and VM. However, inclusion of the extreme high‐range VM values produced a better fit with a polynomial model, likely due to measurement distortions at high humidity levels inherent to the VM's closed‐chamber design. However, Fell et al. [21].reported TEWL values up to a mean of 35 g/m^2^/h, beyond which, we observed the VM anomalies. Their study, though reporting comparable ICCs (0.81 for scars and 0.52 for healthy skin), had a small sample size and limited repeated measurements (two by the first rater, one by the second), limiting the generalizability of their results.

Our findings highlight the importance of selecting an appropriate device based on the anticipated TEWL range. Each device has specific limitations and strengths, for example the VM is a single hand‐held unit whereas the DL is connected to a computer with a wire. In the medium‐range of TEWL (3–15 g/m^2^/h, DL or 8–20 g/m^2^/hr VM), both devices showed excellent intra‐rater and inter‐device reliability, though the VM consistently measured approximately twice the DL values. At low TEWL levels, the VM may be preferable until more clarity is achieved around DL's measurement protocols, specifically the setting of the SD target. In contrast, for high TEWL values, the DL appears more reliable due to the VM's potential saturation limit.

To ensure accuracy and consistency in TEWL measurements, clinicians and researchers should replicate device use within studies and be aware of each device's optimal operating range. The consistency of using a single device remains critical.

### 4.1 Limitations

This analysis has several limitations. For low‐range TEWL, only the DL's 0.2 SD target or 30‐s cut‐off was applied, potentially contributing to variability. Additionally, the order of device application was neither randomized nor protocolised across the three studies. Although three measurements were obtained by alternating each device at the same anatomical site to minimise measurement error, the potential influence of measurement order cannot be excluded. However, given that both devices are associated with minimal physical disturbance to the skin, the effect first device use is likely negligible.

Waiting times for device ventilation were not standardised or formally timed. The VM provides an indicator when ventilation is complete and the device is ready for subsequent measurement, whereas the DL does not; readiness is only apparent upon initiation of measurement. Consequently, the DL required close monitoring during data collection.

Future studies should standardise inter‐measurement intervals and incorporate randomisation and protocolisation of device use to reduce potential sources of bias.

The The high TEWL range was underrepresented, with only 274 data pairs and only 14 data pairs where the DL measured above 40 g/m^2^/hr. Closer examination in this upper range where the DL measures above 40 g/m^2^/hr, is warranted to more precisely define the VM's upper measurement limit as these data points appear to be outliers and do not follow the linear relationship of data below this level of TEWL measurement.

### 4.2 Future Directions

Further research should refine DL's low‐TEWL measurement protocols, particularly with regard to measurement time and SD thresholds. Additionally, calibration of the VM against standardized TEWL outputs > 40 g/m^2^/h (as measured by DL) is necessary to establish its upper functional limit. Given the number of recent reliability studies, a meta‐analysis of ICCs may soon be feasible and would provide more definitive guidance on device selection and reliability.

## 5 Conclusion

This study, leveraging a large and robust dataset, provides important insights into the relative performance of the DL and VM across different TEWL conditions. Our findings underscore the need for device‐specific recommendations based on TEWL range. As measurement reliability varies across the spectrum, understanding these nuances is crucial for selecting the most appropriate device in both clinical and research settings.

## Funding

This study is intended to contribute towards a Doctor of Philosophy in Medicine at the Adelaide Medical School, The University of Adelaide, for the primary author (TK). I (TK), acknowledge the support I have received for my research through the provision of an Australian Government Research Training Program Scholarship.

Study 1 received funding from the Hospital Research Foundation Group, South Australia for investigator time. For study 3, payment of the honorarium to participants and procurement of tape stripping supplies was funded by the Department of Trauma and Surgery, Royal Adelaide Hospital, Adelaide, South Australia.

## Conflicts of Interest

There are no conflicts of interest to declare. All authors have no affiliations with any of the devices, companies and associates, or their competitors, that have been mentioned in this analysis of data.

## Data Availability

The data that support the findings of this study are available from the corresponding author upon reasonable request.

## Appendix 1 Sensitivity Analysis

### 1.1

**TABLE A1:** Intraclass correlation coefficients (ICC) and 95% confidence intervals (CI) for inter‐device reliability where measurements were taken with DermaLab and the VapoMeter. These statistics are presented across the low, medium, and high TEWL ranges, using TEWL values one unit lower than those reported in the manuscript (as measured by the DermaLab). The mean percentage difference, standard deviation (SD), coefficient of variation (CV) and Standard Error of Measurement (SEM) are calculated using the percentage difference between the two devices.

Sample	Mean % difference^a^	n	SD	CV	ICC (95% CI)	Interpretation	SEM
Low range^b^	480.67%	144	337.34%	70.18%	0.1950** (−0.1188, 0.4207)	Poor reliability	302.67%
Medium range^c^	117.61%	1177	69.24%	58.87%	0.9175* (0.9075, 0.9264)	Excellent reliability	19.89%
High range^d^	34.68%	296	34.80%	100.35%	0.6252* (0.5289, 0.7018)	Moderate reliability	20.22%

^a^ *Percentage difference = (VapoMeter value—DermaLab Value / DermaLab Value) x100.

^b^ Low range: DermaLab 0–2; VapoMeter 0–10 (approximately).

^c^ Mid range: DermaLab > 2 – 14; VapoMeter > 10–26 (approximately).

^d^ High range: DermaLab > 14; VapoMeter > 26 (approximately).

* *p*<0.001.

** *p* = 0.098.

**TABLE A2:** Intraclass correlation coefficients (ICC) and 95% confidence intervals (CI) for inter‐device reliability where measurements were taken with DermaLab and the VapoMeter. These statistics are presented across the low, medium, and high TEWL ranges, using TEWL values one unit higher than those reported in the manuscript (as measured by the DermaLab). The mean percentage difference, standard deviation (SD), coefficient of variation (CV) and Standard Error of Measurement (SEM) are calculated using the percentage difference between the two devices.

Sample	Mean % difference^a^	n	SD	CV	ICC (95% CI)	Interpretation	SEM
Low range^b^	267.49%	528	226.05%	84.51%	0.5367* (0.4503, 0.6095)	Moderate reliability	153.86%
Medium range^c^	80.88%	848	39.37%	48.68%	0.9076* (0.8942, 0.9192)	Excellent reliability	11.97%
High range^d^	33.56%	241	37.42%	111.50%	0.5976* (0.4814, 0.6877)	Moderate reliability	23.74%

^a^ Percentage difference = (VapoMeter value—DermaLab Value / DermaLab Value) x100.

^b^ Low range: DermaLab 0–4; VapoMeter 0–12 (approximately).

^c^ Mid range: DermaLab > 4 – 16; VapoMeter > 12–29 (approximately).

^d^ High range: DermaLab > 16; VapoMeter > 29 (approximately).

* *p*<0.001.
